# YOLOv11n-DualPC-Lite: a lightweight, high-precision real-time detection model for maize leaf diseases

**DOI:** 10.3389/fpls.2026.1797083

**Published:** 2026-03-25

**Authors:** Peng Zhou, Qingqing Wang, Min Zhan, Bin Zhu, Kechuan Yi, Chunxia Jiang, Juan Wang

**Affiliations:** 1 Agricultural Equipment Laboratory, College of Intelligent Manufacturing, Anhui Science and Technology University, Chuzhou, Anhui, China; 2 Fruit Industry Equipment Laboratory, College of Engineering, South China Agricultural University, Guangzhou, Guangdong, China

**Keywords:** high precision, lightweight, maize leaf disease, real-time detection, YOLOv11n

## Abstract

To address the challenge of balancing model lightweight and detection accuracy in maize leaf disease detection, as well as the limitations of edge device deployment resources, we propose an enhanced target detection model, YOLOv11n-DualPC-Lite.Firstly, the C2fDualPConv module was designed, integrating PartialConv to replace some C3k2 modules in the backbone and neck networks. This approach enhances feature representation while reducing the number of parameters. Secondly, the Slim-Neck architecture is introduced in the neck network. To improve accuracy without increasing the number of parameters, the VoVGSCSPC_SimAm module enables the new Slim-Neck structure to reduce parameters while strengthening feature representation. Finally, an EfficientHead detection head is introduced that uses an inverted bottleneck MBConv module to improve performance. This significantly reduces computational load while efficiently extracting features. This study constructed a maize leaf disease dataset integrating a publicly available Kaggle dataset and a field-collected dataset from Anhui Science and Technology University’s experimental plots. The dataset includes four categories: Blight, Common_Rust, Gray_Leaf_Spot, and Health. Through techniques such as rotation and gamma correction, the dataset was expanded from 3,876 to 5,165 images for model training and performance validation. Test results show this improved model performs better than other popular lightweight models overall, with a mAP50 score of 90.9%. Meanwhile, the model has only 2.13 million parameters; its computational complexity is reduced to 4.55 G, and the model size is 4.41 MB. Compared with the original YOLOv11n, its mAP50 is 1.9% higher, while the number of parameters is down by 17.8%, computational complexity is cut by 29.3%, and file size is reduced by 15.7%. When run on a Raspberry Pi 5, the model’s detection speed reaches 2.3 FPS, an increase of 27.8%. This model achieves a good balance between detection accuracy and lightweight performance for maize leaf diseases, providing an efficient and practical method for real-time crop disease monitoring.

## 1 Introduction

As one of the world’s three major food crops, maize output and quality directly affect global food security and the steady growth of the farm economy (Cheng et al., 2025; Pan et al., 2025). In China, maize cultivation is widespread, serving as a core raw material for feed production, industrial processing, and daily household consumption. Its yield exerts a profound influence on the agricultural supply chain (Gao et al., 2023). However, in its growth cycle, maize is very easy to get various diseases, including blight, common rust and grey leaf spot (Dai et al., 2023). These diseases are characterized by rapid transmission, severe damage, and high concealment. Under good temperature and moisture conditions, they can spread quickly, cutting down the maize leaves’ photosynthesis ability and leading to nutrient loss. In serious cases, they result in yield reductions or even total crop failure (Berger et al., 2014).

Traditional maize leaf disease detection mainly depends on manual field checks and practical experience, along with chemical testing, microscopic observation and other methods (Song et al., 2023; Li et al., 2024; Sun et al., 2024). Manual inspection has low costs and simple operation, yet it has obvious drawbacks. Results are highly subjective, based on personal experience, and are easy to cause missed or wrong diagnoses (Liu et al., 2022). Its efficiency is low, making it unable to monitor large-scale planting areas. Early disease symptoms are hard to spot in time, often leading to the loss of the best prevention and treatment periods (Wang et al., 2025a). Traditional machine learning methods, such as Support Vector Machines (SVM) and Random Forests, require manual design of features like lesion color and shape (Nisar et al., 2020; Ahmad Loti et al., 2021; Liu and Wang, 2021), resulting in poor generalization capabilities and a marked decline in performance under complex field conditions (Arnal Barbedo, 2013; Domingues et al., 2022). For instance, Padol et al. employed K-means clustering combined with SVM classification, achieving an accuracy of 88.89% across 137 images (Padol and Yadav, 2016); Li et al. used principal component analysis and probabilistic neural networks to detect rice diseases, achieving an accuracy of 95.65% (Bo et al., 2009). However, these methods depend heavily on feature extraction and segmentation. This makes them hard to apply in the universal detection scenarios for multiple maize diseases (Karthik et al., 2020; Shoaib et al., 2023).

The rapid development of deep learning technology has empowered computer vision tasks such asimage classification and object detection. It has also opened new avenues for the intelligent detection of plant diseases and pests (Wang et al., 2019; Yang et al., 2022; Tian et al., 2023; Zhong et al., 2024). Object detection algorithms, as the core technology in this field, are categorized into two-stage and single-stage approaches (Chu et al., 2024). Two-stage algorithms, exemplified by R-CNN (Dong et al., 2023), Fast R-CNN (Wang et al., 2017), and Faster R-CNN, achieve detection through the process of ‘generating candidate regions + precise classification and localization’, demonstrating significant accuracy advantages. For instance, Liu et al. achieved 95.48% accuracy in detecting rice stem and leaf diseases using Faster R-CNN (Liu et al., 2020). However, the high computational demands and lengthy processing times associated with multi-step detection make it challenging to adapt to the requirements of real-time field detection (Wang et al., 2025b). Single-stage algorithms, exemplified by the YOLO series and SSD, achieve faster detection speeds by simultaneously performing localization and classification in a single forward pass, making them better suited to real-time agricultural detection scenarios (Zhai et al., 2020; Jiang et al., 2022). In recent years, research on enhancing YOLO series models has garnered significant attention: Lv et al. incorporated residual units into YOLOv3 to strengthen small object location information extraction capabilities (Lv et al., 2022); Zhang et al. proposed a context-guided module based on YOLOv4, achieving a 7.2% improvement in average precision (Zhang et al., 2022); Ma et al. proposed an improved YOLOv5s-SNV2-GSE model, which achieves real-time and efficient detection of lychee pests and diseases on resource-constrained devices by replacing the backbone network, introducing an attention mechanism, and optimizing the loss function (Ma et al., 2025); Liu et al. constructed the lightweight YOLO-DCPG architecture, combining dual-channel pooled gated attention with a lightweight backbone network to effectively enhance detection performance and deployment efficiency for dense small agricultural pests (Liu et al., 2025); Li et al. proposed the ADQ-YOLOv8m model, which significantly improved detection accuracy and robustness for sugarcane diseases in complex environments through dynamic feature enhancement and loss function optimization (Li et al., 2025).

In the field of maize leaf disease detection, related research has made notable progress. AhilaPriyadharshini et al. adopted deep convolutional neural networks to classify maize leafdiseases. The method achieved an accuracy of 97.89% (Ahila Priyadharshini et al., 2019). Yang et al. proposed the YOLO-SDW algorithm, introducing SPD-Conv and Wise-IoU V3 loss functions to enhance model adaptability (Yang et al., 2024a). Yang et al. enhanced maize leaf disease detection performance in complex field environments by introducing Attention and Slim-neck modules to improve YOLOv8 (Yang et al., 2024b). Gan X et al (Gan et al., 2025) proposed the YOLOv8-DBW algorithm, which achieves model lightweighting while improving detection accuracy by introducing DSConv modules into the backbone network, adopting a BiFPN bidirectional feature pyramid structure, and incorporating the Wise-IoU loss function. Meng et al (Meng et al., 2025). proposed the YOLO-MSM algorithm, which integrates multi-scale variable kernel convolution (MKConv), SK attention mechanism, and MPDIoU loss function. With a model size of only 5.4 MB, it achieves significant reductions in parameters and computational complexity. This lightweight design makes it highly suitable for mobile deployment. However, current detection models still face several challenges. First, most high-precision models come with a large number of parameters and high computing complexity. They require plenty of computing resources, making it hard to install them on terminal devices such as Raspberry Pi. Second, while certain improved algorithms have boosted disease recognition performance in complex field environments, there is still considerable room for improvement in overall detection precision. Third, while some lightweight models effectively reduce computational complexity, they sacrifice inference accuracy, highlighting the challenge of balancing precision and compactness. In addition, issues such as the difficulty in obtaining disease image datasets, insufficient sample size, and low detection accuracy for small target diseases further restrict the practical implementation capabilities of the model.

Therefore, developing a maize leaf disease detection model that balances high accuracy, lightweight design and real-time performance is an urgent need for promoting the practical use of smart agricultural monitoring. As a lightweight YOLO model, YOLOv11n has inherent advantages in terms of fewer parameters and higher running speed (Ganapathy et al., 2025); however, its feature detection and multi-scale merging ability still have room for improvement in complicated farmland environments. Taking this into account, this paper proposes YOLOv11n-DualPC-Lite, a lightweight and high-precision detection model based on YOLOv11n. The main contributions of this research are listed as follows:

1. Proposes an enhanced object detection model, YOLOv11n-DualPC-Lite. The backbone and neck networks incorporate the C2fDualPConv module, which integrates PartialConv to reduce the parameter count while preserving expressive power substantially. Further, the VoVGSCSPC_SimAm module, incorporating SimAm attention, is introduced to construct the Slim-Neck architecture. This enhances multi-scale feature fusion capabilities without increasing computational burden. Concurrently, an EfficientHead detection module based on MBConv is adopted, substantially reducing inference computational requirements. The improved model significantly enhances detection accuracy while amplifying its lightweight advantages.
2. Verifying the model’s general applicability and practical value in actual agricultural settings. This study built a dataset by combining public and field-gathered data, which was then data-augmented and quality-optimized. Thorough testing of the proposed model was carried out on this dataset, showing good performance in multi-type disease detection work. What’s more, the model was successfully run on a Raspberry Pi 5 edge device, realizing stable, real-time detection and providing a workable technical method for field disease monitoring.

## 2 Materials and methods

### 2.1 Dataset introduction

**Figure 1:**
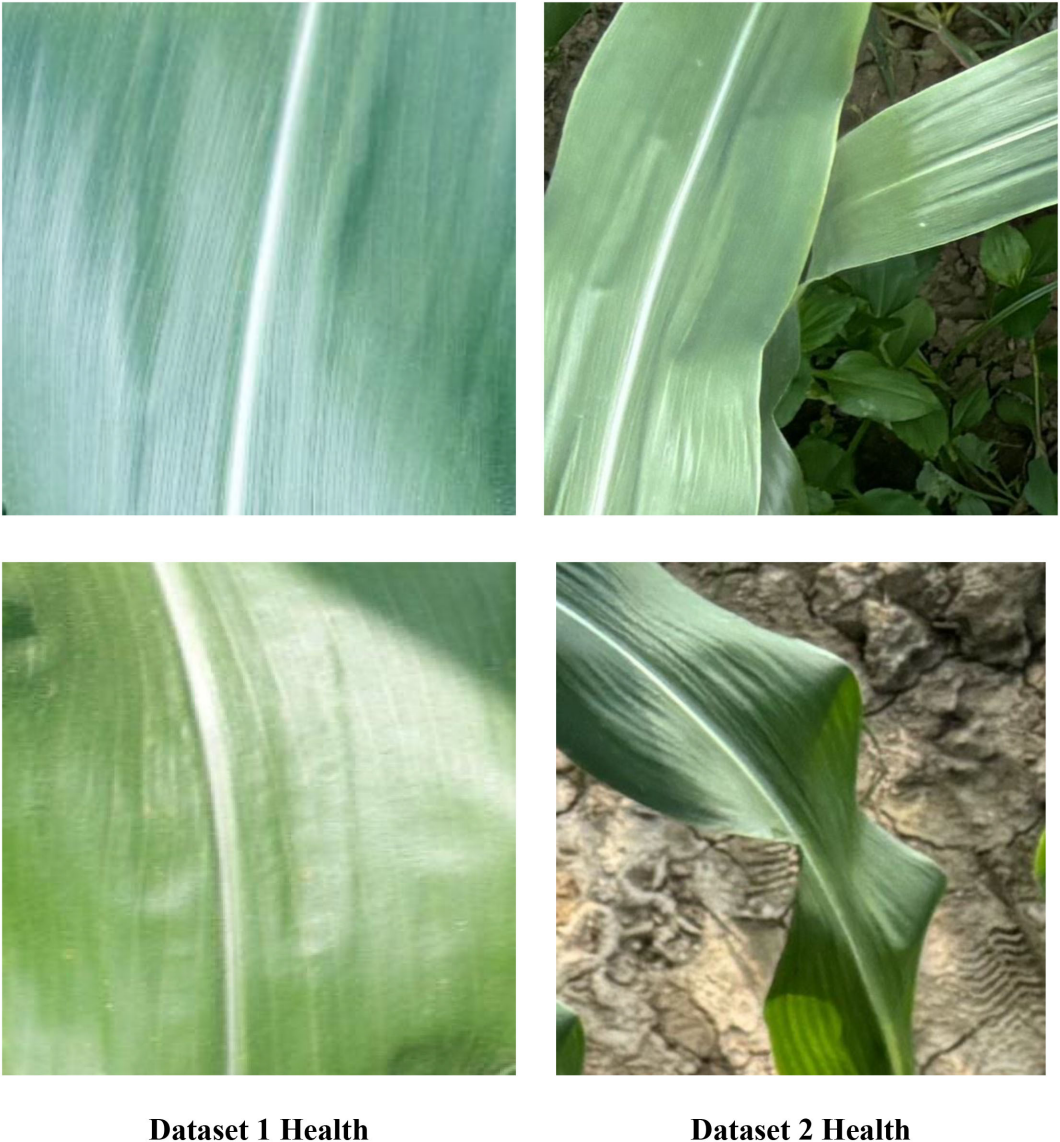
Healthy images.

**Figure 2:**
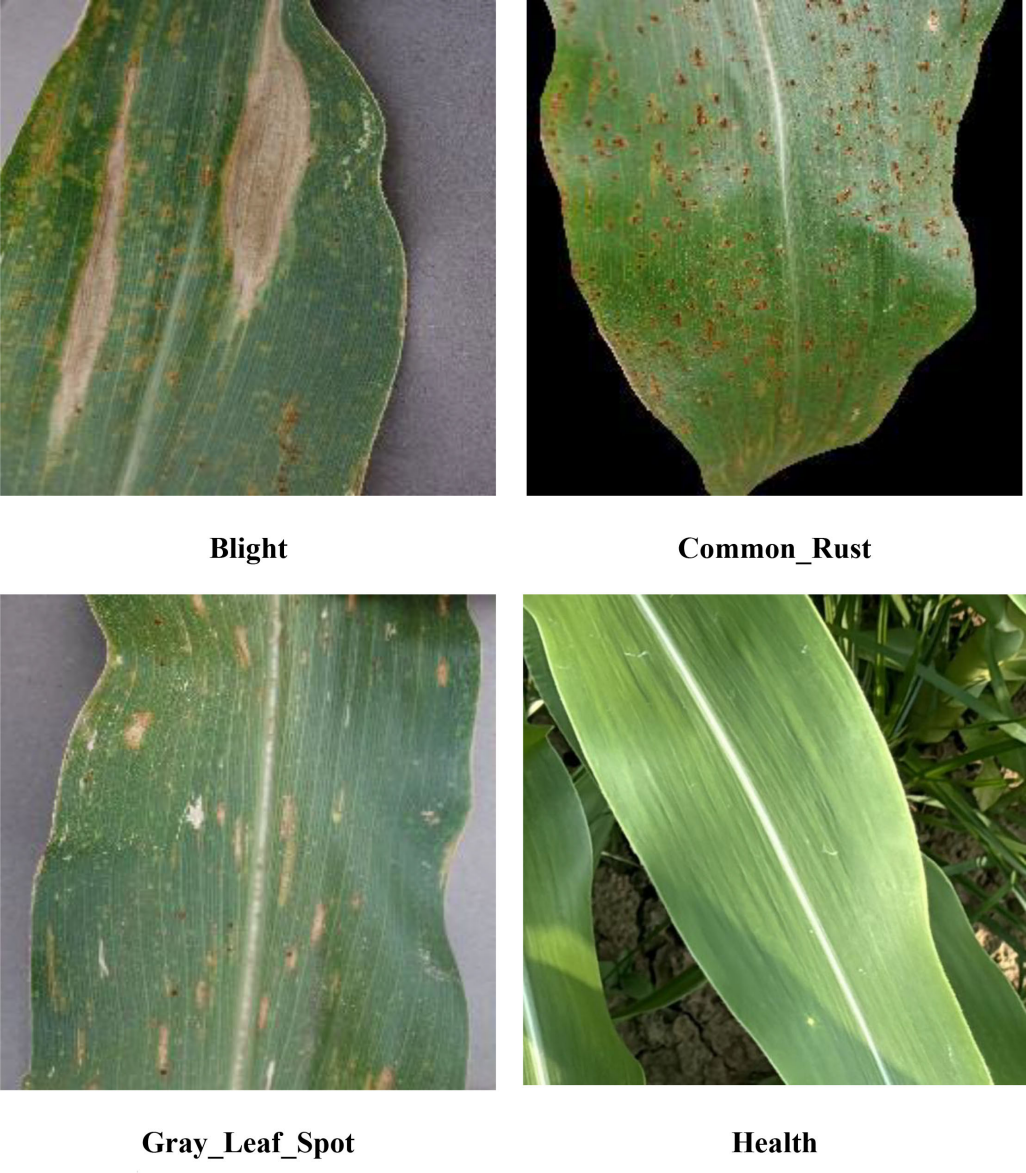
Sample images from the dataset.

The dataset constructed in this study comprises two datasets: Dataset 1 from the Kaggle data website (https://www.kaggle.com/datasets/hendriyunuswijaya/maize-leaf-disease) and Dataset 2 collected from the experimental field at Anhui Science and Technology University in Chuzhou City, Anhui Province. Dataset 1 contains a total of 4,188 images, including 1,162 images in the Health category. All images depict only specific regions of healthy maize leaves without complex backgrounds, which differs significantly from actual field conditions. This discrepancy may interfere with model training and reduce the model’s real-time recognition capabilities in real-world scenarios. Therefore, only 250 images in the Health category were retained, while all 3,026 images in the other categories were fully preserved. Dataset 2 contains a total of 600 healthy images. These images were captured using a mobile phone in JPG format between 9:00 AM and 12:00 PM on July 10, 2025, during clear weather conditions. The images depict healthy maize leaves at the tasseling stage, collected from the experimental fields of Anhui University of Science and Technology in Chuzhou City, Anhui Province. As shown in **Figure 1**, the healthy maize leaf images were selected from Dataset 1 and Dataset 2. This dataset comprises four categories: Blight, Common_Rust, Gray_Leaf_Spot, and Health, as illustrated in **Figure 2**.

### 2.2 Dataset construction

**Figure 3:**
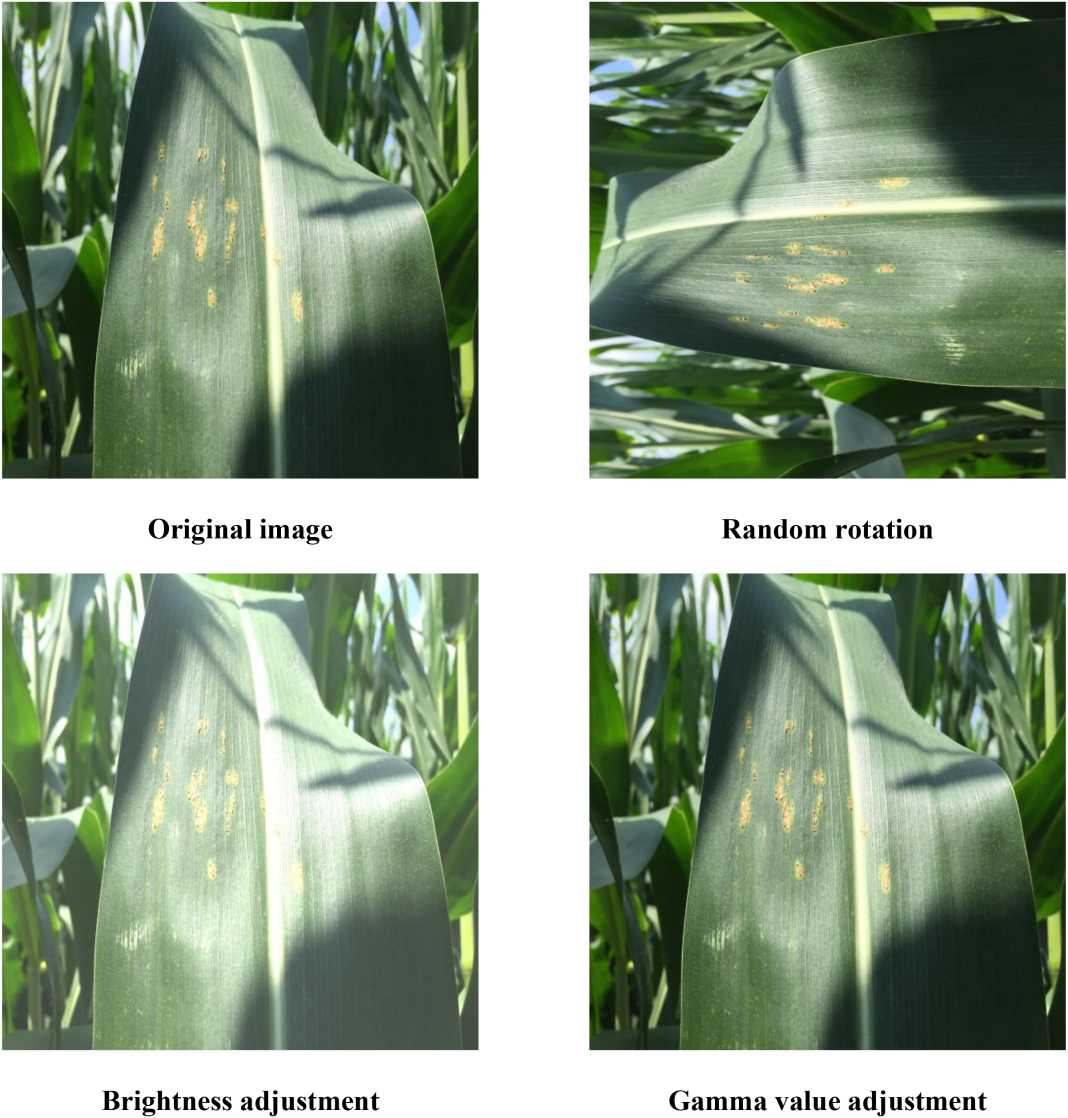
Examples of images augmentation.

To expand the dataset size and enhance the model’s generalization capability and detection accuracy, this study employs rotation, brightness adjustment, and gamma correction methods to augment the dataset, as shown in **Figure 3**. Rotation simulates the diverse spatial orientations of maize leaves in the field. Brightness adjustment can simulate different lighting conditions. Gamma correction optimizes the uniformity of image brightness distribution, enhancing image quality. The aforementioned methods are all classic and effective data augmentation techniques in the context of plant leaf disease identification, characterized by their simplicity of implementation and ability to enhance model robustness.

After augmentation, the dataset was expanded from the original 3,876 images to 5,165 images. The augmented leaf sample images are shown in **Figure 3**. All images were annotated using the LabelImage tool, with annotation files saved in TXT format. The final dataset was divided into training, validation, and test sets in an 8:1:1 ratio, as shown in **Table 1**.

**Table 1:** Maize leaf disease dataset.

Categories of diseases	Training set/sheet	Verification set/sheet	Test set/sheet	Total/sheet
Blight	916	120	110	1146
Common_Rust	1044	140	122	1306
Gray_Leaf_Spot	917	120	110	1147
Health	1252	157	157	1566
All	4129	537	499	5165

### 2.3 Improved YOLOv11n model

YOLOv11 is an object detection model released by the Ultralytics team in 2024, available in five scaled variants: n, s, m, l, and x. Compared to YOLOv8, its architecture has targeted upgrades: it adds C3k2 and C2PSA modules as well as a new detection head. This keeps detection performance steady while further boosting computing speed and feature expression ability.

Among these, the C3k2 module inherits from C2f, adhering to the CSP principle: replacing a large convolution with two small convolutions to reduce computational complexity, while simultaneously enhancing the model’s generalization capability through parameter tuning and the C2f wrapping of C3k. The C2PSA module encapsulates the PSABlock within C2f. By combining attention mechanisms with FFN residual connections, it improves the module’s stacking ability, strengthens spatial connections and feature dimensions, and shows better ability to detect small and blocked objects. The classification branch of the new detection head abandons simple 1×1 convolutions in favor of a DWConv+PointwiseConv combination, enhancing nonlinear representation and structural decoupling. When paired with the attention module, this strengthens detection in high-response regions, thereby improving adaptation to complex scene recognition. YOLOv11 maintains YOLOv10’s NMS-free training strategy to build an end-to-end architecture, improving model performance and deployment flexibility. Notably, YOLOv11n adopts a low-complexity, small-scale feature design. This greatly reduces computing resource usage and speeds up running speed, making it especially suitable for real-time agricultural monitoring situations.

**Figure 4:**
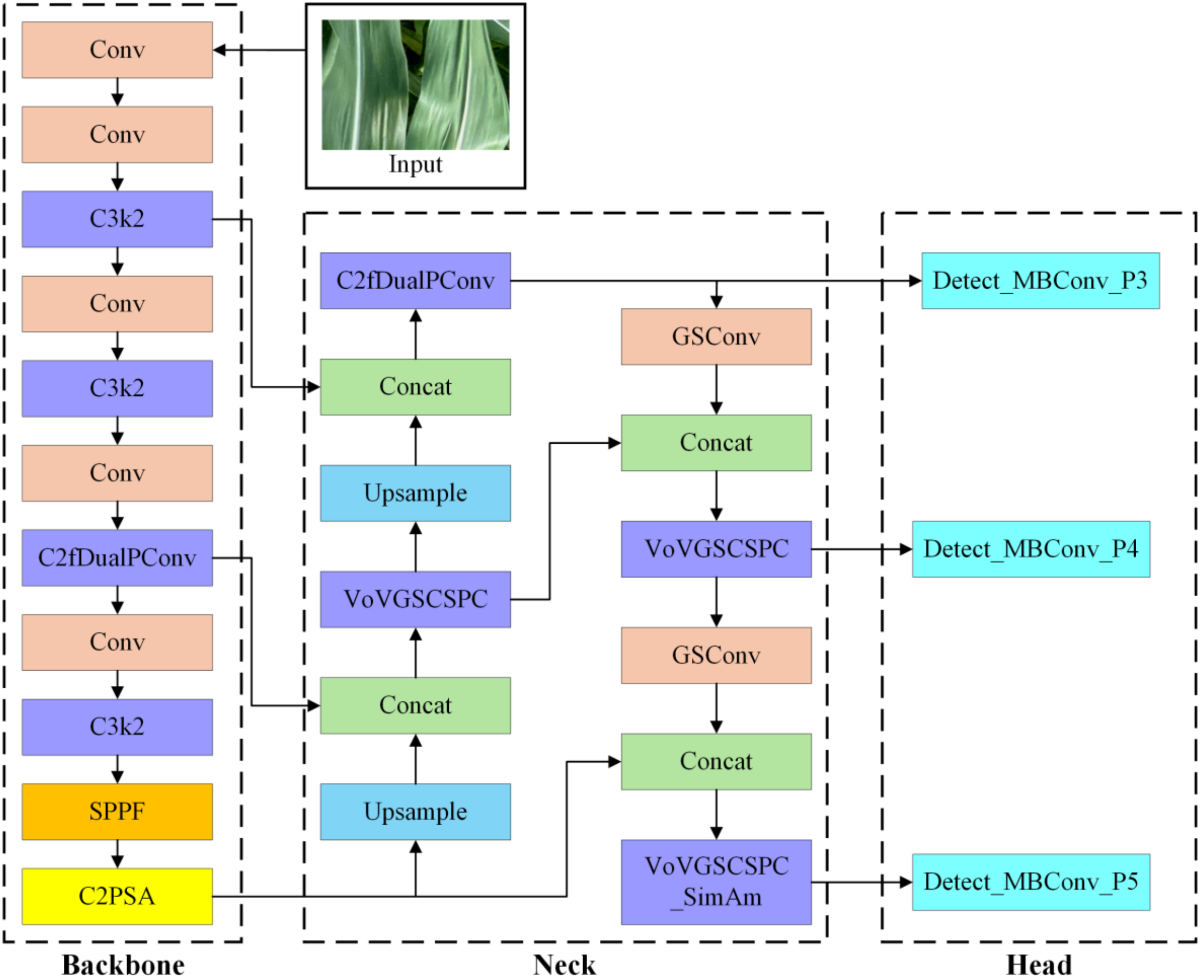
Model network structure diagram.

Although the YOLOv11n model shows good overall performance in feature extraction and model size, it still has computing constraints when used on embedded devices. To boost feature extraction speed and disease recognition accuracy for different maize leaf diseases, this study puts forward an improved model named YOLOv11n-DualPC-Lite, which is based on YOLOv11n. The main improvements are listed below:

1. A C2fDualPConv module incorporating a Partial Conv mechanism has been designed for both the backbone and neck networks. The internal branch modules can be flexibly switched via the c3k parameter, enabling the replacement of the C3k2 module with different branch modules. This approach enhances feature representation while reducing the number of parameters.
2. A Slim-Neck architecture is introduced in the neck network. To enhance accuracy without increasing the number of parameters, the VoVGSCSPC_SimAm module enables the new Slim-Neck structure to improve feature representation while reducing the number of parameters.
3. A lightweight and efficient detection head, EfficientHead, is introduced. By leveraging the Mobile Bottleneck Convolution (MBConv) module from EfficientNet to refine the detection head, it achieves efficient feature extraction while substantially reducing computational load. The overall model architecture is illustrated in **Figure 4**.

#### 2.3.1 C2fDualPConv module

To further reduce the model’s parameters and calculation cost while keeping detection accuracy unchanged, this paper designs the C2fDualPConv module. It is used in both the backbone and neck networks of YOLO11n, balancing lightweight design and feature expression ability. The C2fDualPConv module takes the C2f module as its basic framework. C2f is an efficient, fast CSP bottleneck structure that improves computing efficiency through feature division and multi-branch combination. We have targeted replacing the core feature extraction units in C2f: the original C2f Bottleneck module is replaced with the CSPPC_Bottleneck module, building a lightweight but highly effective feature-extraction branch. After the ‘1×1 convolution - channel splitting’ process in C2f, this module employs c3k parameter switching to configure branch strategies differentially across network levels. The C2fPConv branch is activated in the backbone network to strengthen deep feature capture, while the PartialConv branch is used in the neck network to further cut calculation cost.

**Figure 5:**
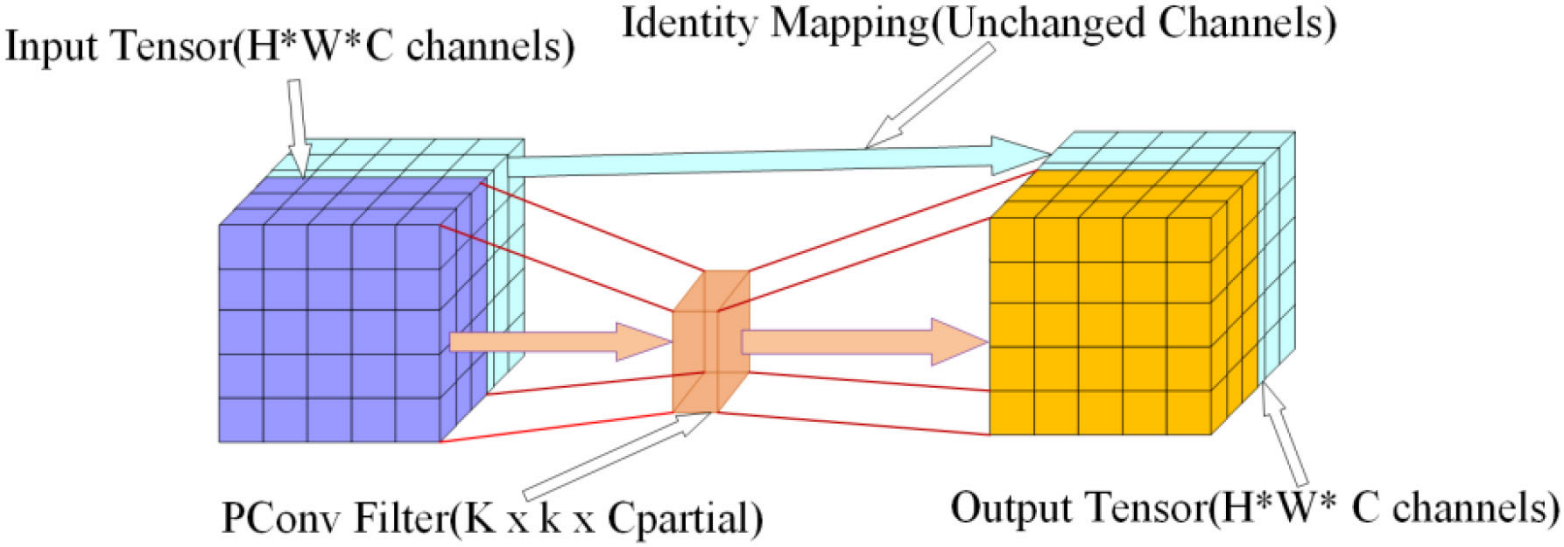
Partialconv structural diagram.

The core of CSPPC_Bottleneck lies in its DualPConv (Chen et al., 2023) architecture, comprising two stacked Partialconv modules. Partialconv employs a ‘channel partial convolution’ strategy: it proportionally divides the input feature’s channels into convolutional and non-convolutional channels, performing 3×3 convolutions only on a subset of channels rather than executing full convolutions across all channels, as illustrated in **Figure 5**. This approach preserves the spatial feature-extraction capability of 3×3 convolutions while reducing redundant computations via channel decoupling, thereby achieving computational cost compression.

**Figure 6:**
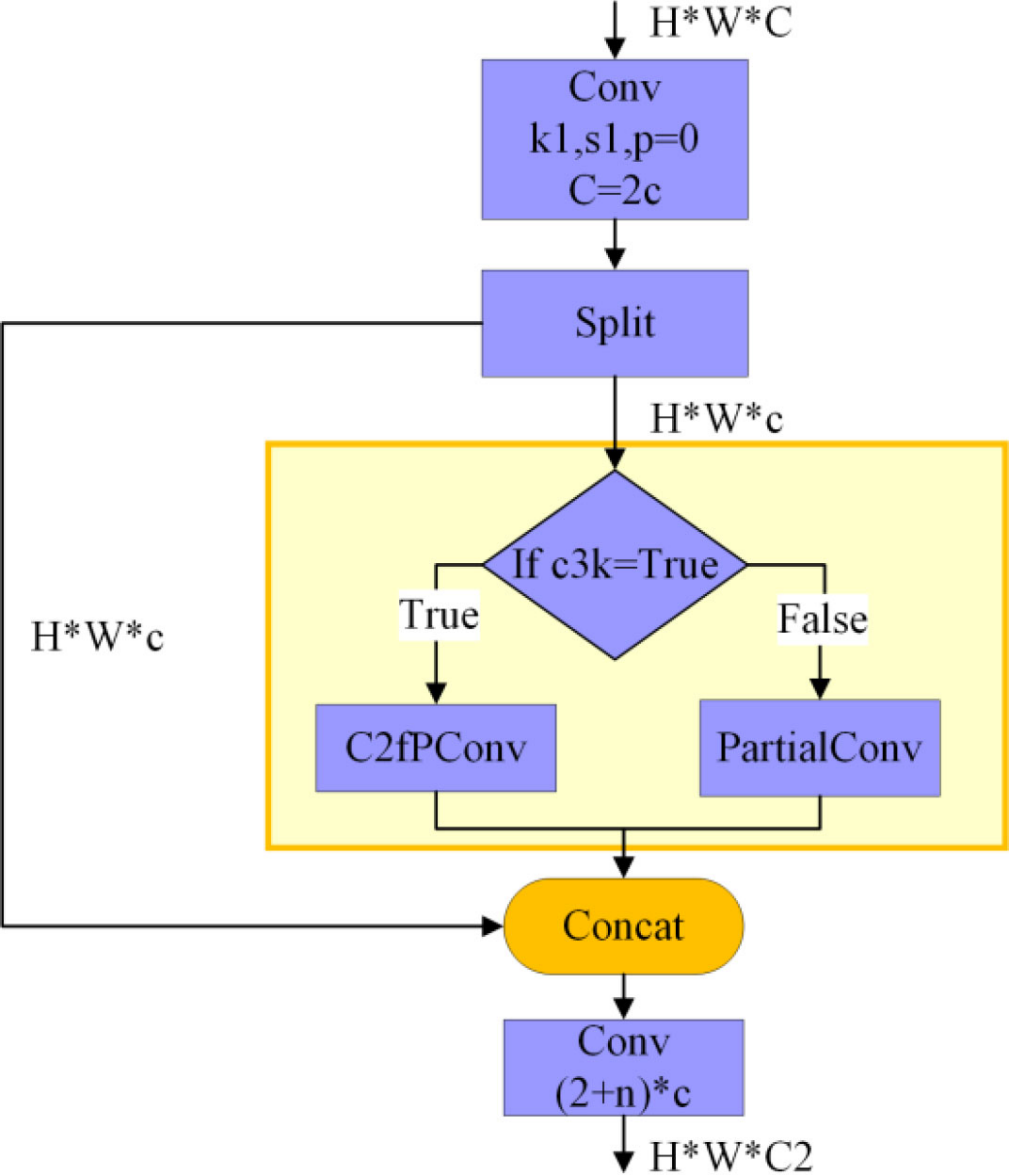
C2fDualPConv module structural diagram.

Compared to traditional C3 and C2f modules, the C2fDualPConv architecture demonstrates significant performance advantages. This architecture introduces a partial-channel convolution mechanism that effectively avoids redundant computations associated with full-channel convolutions. Consequently, it achieves dual reductions in both model parameters and computational complexity. Simultaneously, the C2fDualPConv architecture inherits the multi-branch feature-fusion logic of the C2f module, ensuring effective feature integration. The dual partial convolution stacking structure further enhances the model’s ability to extract fine-grained features, markedly enriching feature representation. The synergistic effect of these characteristics enables the model to maintain or improve detection accuracy while reducing computational overhead. The specific structure of C2fDualPConv is illustrated in **Figure 6**.

#### 2.3.2 Slim-neck architecture and VoVGSCSPC_SimAM module

**Figure 7:**
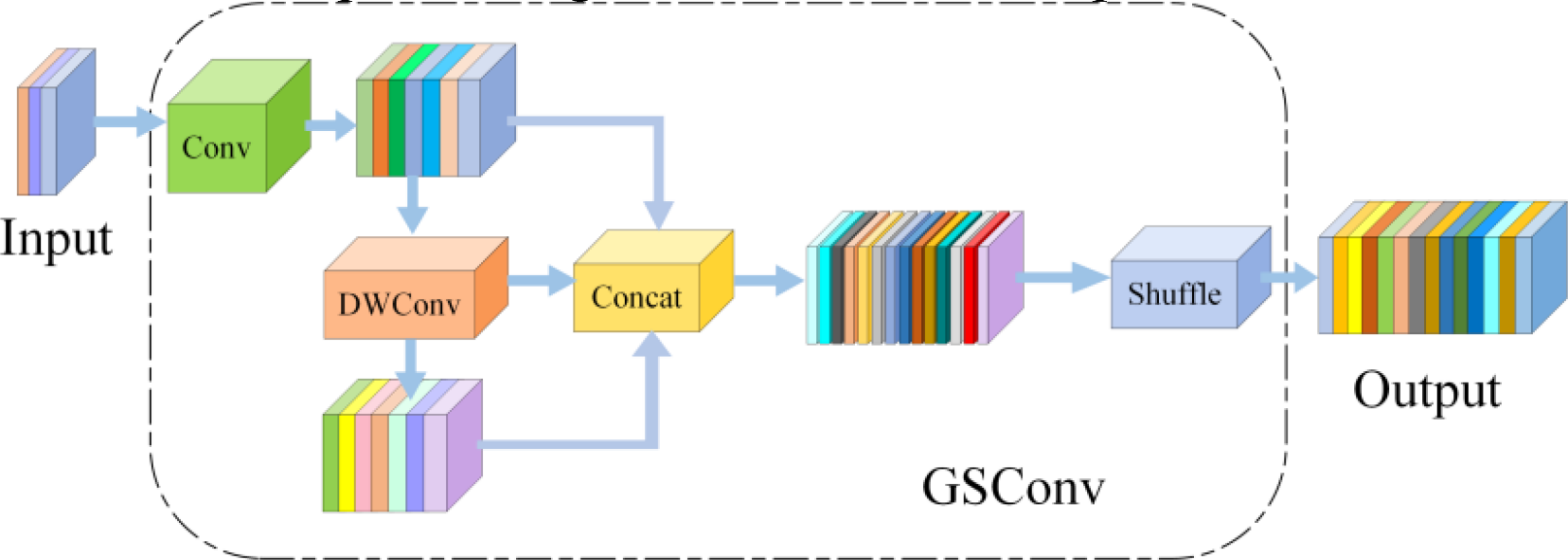
GSConv structural diagram.

**Figure 8:**
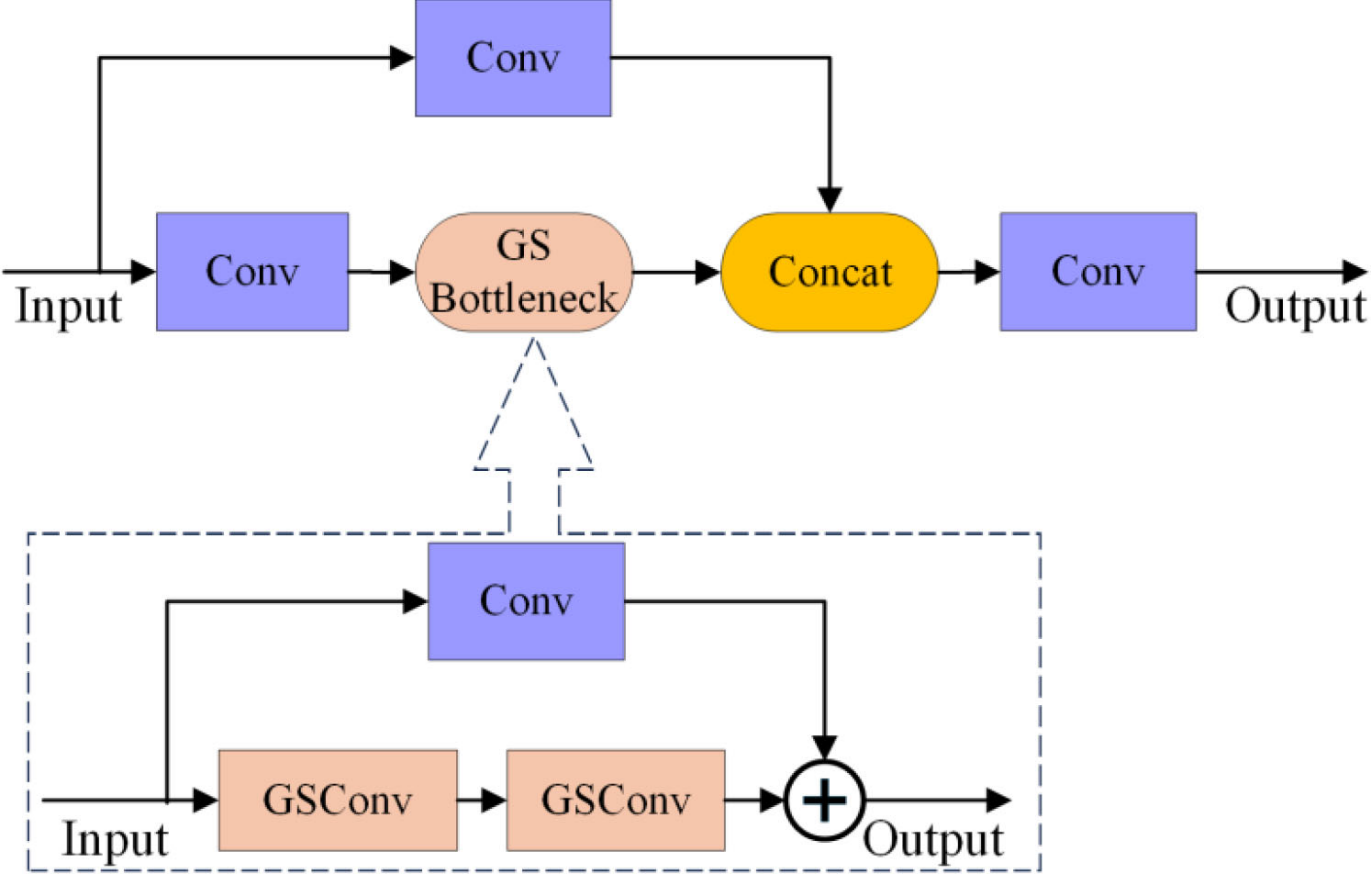
VoVGSCSPC structural diagram.

Slim-Neck is a lightweight neck architecture designed for reduced computational demands, comprising a core structure of GSConv (illustrated in **Figure 7**) and VoVGSCSPC (depicted in **Figure 8**) (Li et al., 2005). GSConv employs a design workflow of ‘standard convolution → depthwise separable convolution → channel reshuffling’. First, a small set of standard convolution kernels is used to extract cross-channel features and spatial information. Subsequently, computational costs are substantially reduced by using depthwise separable convolutions. Finally, channel reshuffling operations facilitate information fusion across channels, mitigating the inherent channel isolation in depthwise separable convolutions. This module replaces the conventional convolution modules in the original YOLO series neck networks. VoVGSCSPC further reduces computational cost beyond GSConv by employing a dual-path feature-extraction logic: Path One performs feature extraction directly via standard convolutions. In contrast, Path Two processes features via standard convolutions followed by the GS Bottleneck (designed based on GSConv). Both feature streams are concatenated and integrated via convolution for the final output. Both GSConv and VoVGSCSPC substantially reduce computational load while preserving sufficient feature representation capability.

**Figure 9:**
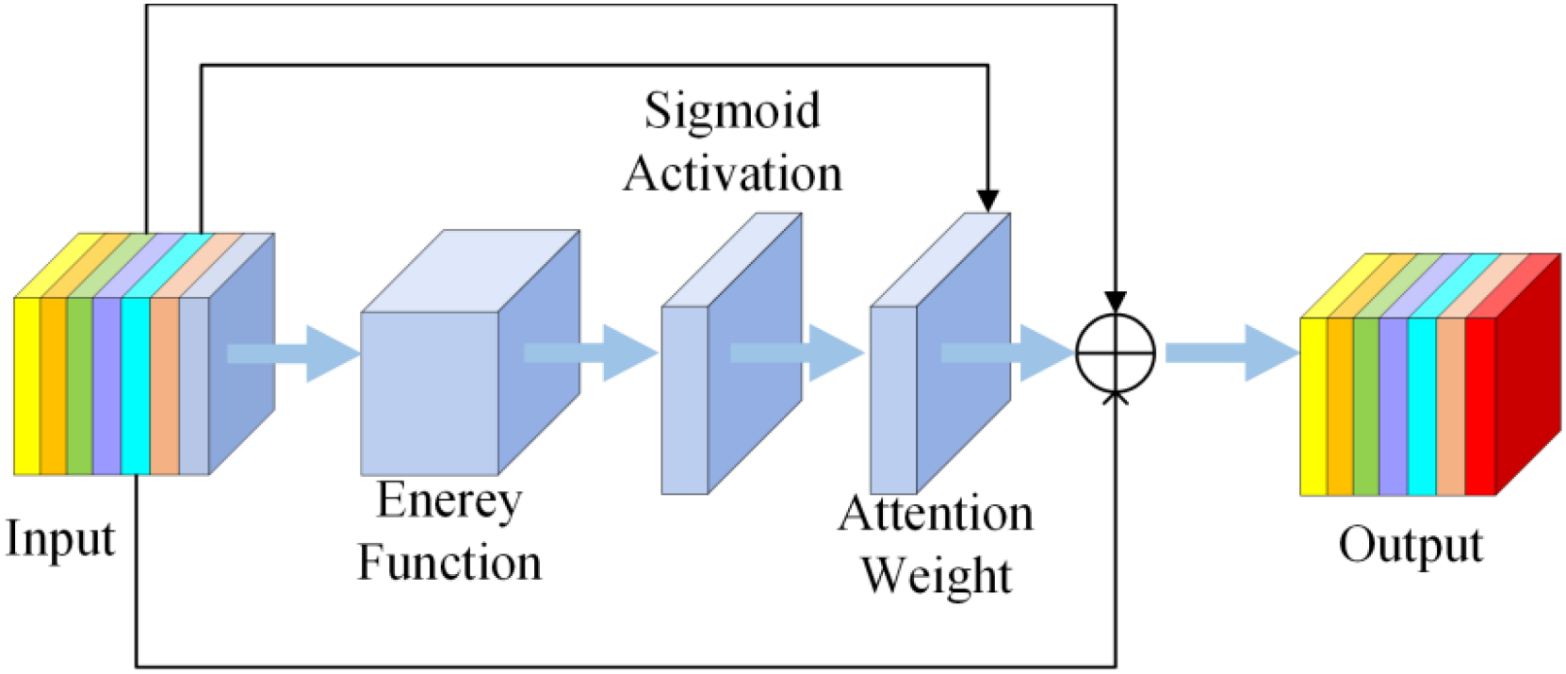
SimAM structural diagram.

**Figure 10:**
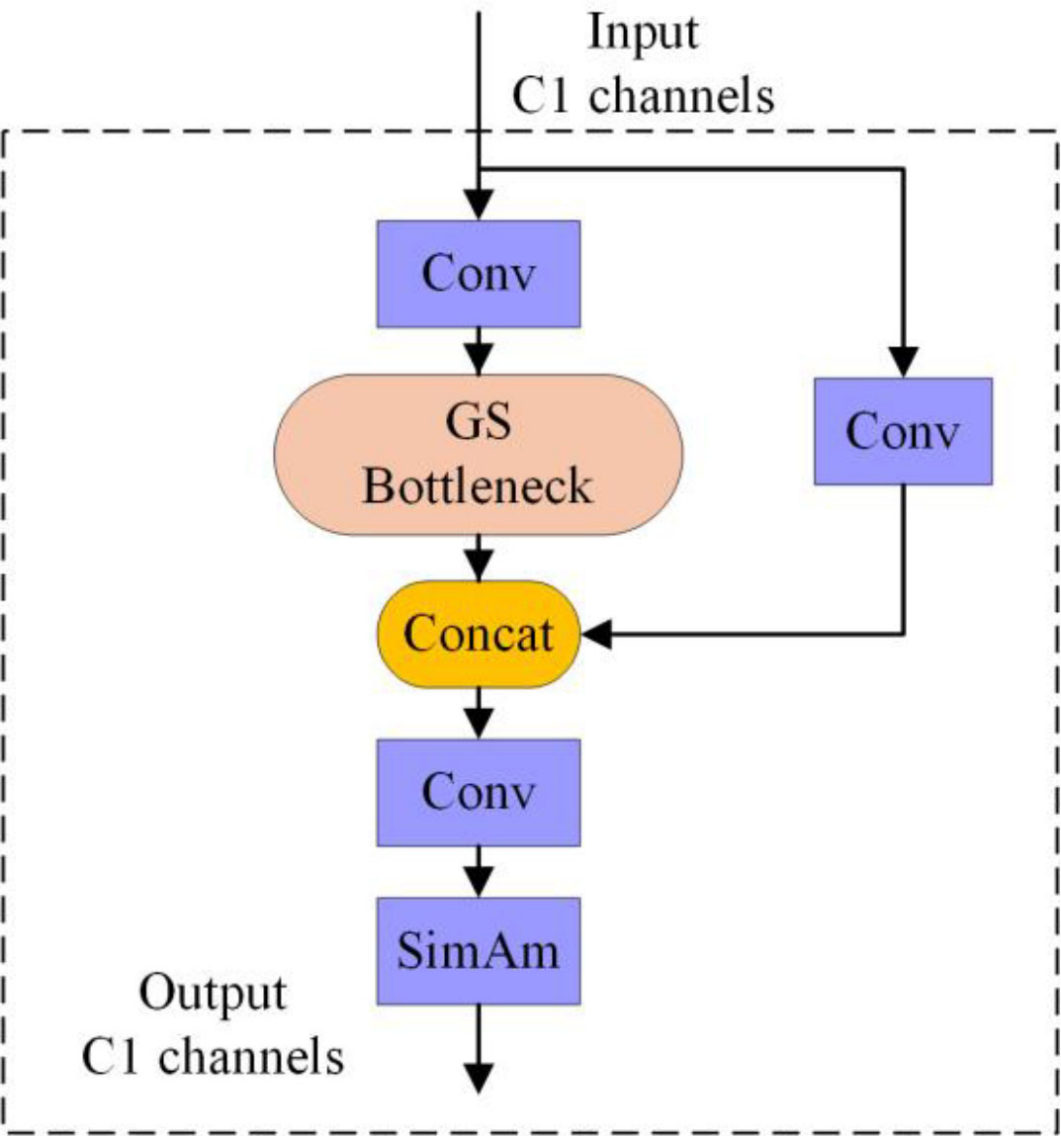
VoVGSCSPC_SimAM structural diagram.

To enhance accuracy without increasing the number of parameters, this paper introduces the Slim-Neck architecture and designs the VoVGSCSPC_SimAM fusion module as an attention-enhanced variant of the VoVGSCSPC module. The SimAM architecture is illustrated in **Figure 9**, while the VoVGSCSPC_SimAM fusion module architecture is depicted in **Figure 10**. This module embeds a parameter-free SimAM attention unit (Yang et al., 2021) at the output of VoVGSCSPC. Computing the mean-squared deviation of feature map positions within the feature space generates an adaptive weight mask. After Sigmoid activation, this mask weights the feature map, thereby enhancing key features and suppressing redundant information without introducing additional parameters. This module is specifically tailored for the P5 large-scale feature layer of YOLO11n, enabling targeted improvements in considerable object detection accuracy. It is also compatible with GSConv’s channel shuffling mechanism. Leveraging the inherent branch-fusion structure of VoVGSCSPC, along with its newly introduced attention enhancement mechanism, it aligns with lightweight, efficient design objectives without increasing the number of parameters.

#### 2.3.3 EfficientHead detection head

**Figure 11:**
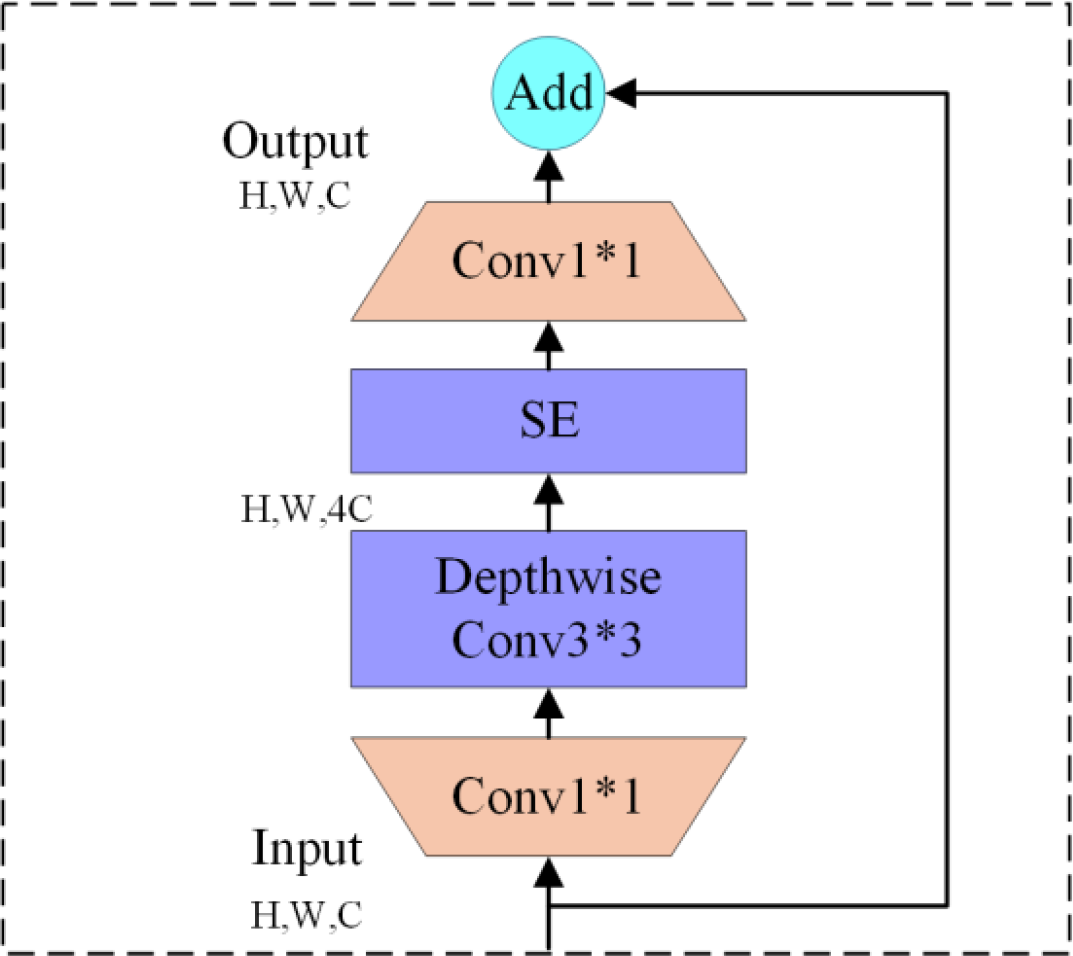
EfficientHead structural diagram.

EfficientHead is a lightweight, high-performance detection head based on improvements to the YOLOv8 head (Tan and Le, 2019). The core design of this detection head adheres to the principle of balancing precision and efficiency. It adopts a decoupled architecture for regression and classification branches. In the regression branch design, two stacked MBConvBlock layers replace the traditional convolutional structure. This branch performs feature transformation through a specific pipeline: ‘channel expansion convolution → depth separable convolution → optional SE attention mechanism → channel projection convolution’. This chain design reduces computational load while preserving fine-grained image features. The regression branch uses drop-connect regularization to mitigate overfitting. Residual connections within the branch activate on demand, ensuring effective feature reuse. The classification branch adopts a two-layer stacked structure that combines depthwise separable convolution with standard convolution. This structure boosts channel feature fusion while lowering the model’s parameter number, making it well-suited for classification tasks. The overall structure of EfficientHead is shown in **Figure 11**.

This architecture retains the core strengths of the YOLOv8 detection head while introducing a DFL (Distributed Focus Loss) integration module to enhance bounding-box regression accuracy. It combines auto-filling with dynamic anchor generation strategies to adapt to multi-scale feature maps, while accelerating model convergence through targeted bias initialization. In short, while maintaining the original detection accuracy, it greatly cuts computing cost by simplifying module design (lightweight reconstruction), striking a good balance between detection precision and running speed in object detection tasks.

### 2.4 Experimental apparatus and parameter settings

The training platform setup used in this study is as follows: the hardware includes 32GB RAM, an Intel i7-14700KF CPU, and an NVIDIA GeForce RTX 3090 Ti GPU (24GB). The software runs on Windows 10, with all programs running on Python 3.10.15 and PyTorch 2.4.1, and CUDA 11.8 is used for faster training. The training parameters are set as follows: image size 640×640, batch size 32, initial learning rate 0.01, and training lasts for 200 iterations. All models are trained from scratch, with no pre-trained weights applied.

### 2.5 Evaluation indicators

In maize leaf disease detection, balancing model complexity and detection accuracy is the core optimization goal. The key metrics for assessing model lightweighting mainly include floating-point operations (FLOPs), total parameters, and model size. These values directly show how efficiently the model runs and how many resources it uses when deployed on hardware devices. Model detection accuracy is measured with core indicators like Intersection over Union (IoU), precision (P), recall (R), average precision (AP), and mean average precision (mAP). Accordingly, this study chooses precision, recall, and mAP as the main metrics to evaluate detection performance. Meanwhile, it uses total parameters and billion-scale floating-point operations (GFLOPs) to measure model complexity.

(1) $$\left\{\begin{array}{l} \text{P}=\frac{\text{TP}}{\text{TP}+\text{FP}}*100\%\\ \text{R}=\frac{\text{TP}}{\text{TP}+\text{FN}}*100\%\\ {\text{AP}}_{\text{k}}=\frac{1}{\text{M}}\sum_{\text{L}=1}^{\text{M}}{\text{P}}_{\text{k}}(\text{L})\Delta \text{R}(\text{L})\\ \text{mAP=}\frac{1}{\text{K}}\sum_{\text{k}=1}^{\text{K}}{\text{AP}}_{\text{k}} \end{array}\right\}$$

In Equation (1), TP denotes the detection results where the intersection-over-union (IoU) between the model-detected bounding box and the actual lesion target is ≥ the set threshold, and the category label matches the true lesion category—that is, the number of correctly identified lesion targets. FP denotes false positive detection results, including cases where the IoU between the detection box and the actual lesion falls below the threshold, category labels are incorrect, or healthy leaf areas are misclassified as lesions. FN denotes the number of actual maize leaf disease lesions not detected by the model with any bounding box meeting both the IoU and category matching conditions, representing the number of missed disease lesions. K represents the total number of maize leaf disease categories (4). M denotes the number of recall rate intervals. Pk (L) indicates the average precision of category k within recall rate interval L. ΔR (L) is the length of recall rate interval L.

## 3 Experimental results and analysis

### 3.1 Performance analysis of the C2fDualPConv module

To verify the efficacy of the proposed C2fDualPConv module for object detection, ablation experiments were conducted using YOLOv11n as the baseline model. Specifically, four comparative configurations were designed for performance benchmarking: the original YOLOv11n, YOLOv11n-C2fPConv, YOLOv11n-PConv, and the proposed YOLOv11n-C2fDualPConv. All other components remained unchanged to ensure fair comparison. Experimental results are presented in **Table 2**.

**Table 2:** Comparison of the performance of four module configurations.

Model	Precision (%)	Recall (%)	mAP50 (%)	mAP50-95 (%)	PLOPs (G)	Parameter (M)	Model size (MB)
YOLOv11n	87.1	86	89	68.1	6.44	2.59	5.23
YOLOv11n-C2fPConv	89.2	84.5	88.8	67.2	6.25	2.55	5.15
YOLOv11n-PConv	90.1	84.9	89	68.3	6.21	2.54	5.12
YOLOv11n-C2fDualPConv	89.5	86.3	89.6	68.1	6.23	2.55	5.14

Experimental results show that different modules have distinct performance advantages. The proposed YOLOv11n-C2fDualPConv achieves the best overall performance, with mAP50 and Recall of 89.6% and 86.3%, respectively. In terms of resource usage, this model’s PLOPs (6.23G), parameter count (2.55M) and model size (5.14MB) lie exactly between YOLOv11n-C2fPConv and YOLOv11n-PConv. This result indicates that the C2fDualPConv module can effectively balance lightweight needs and detection accuracy. It not only boosts feature extraction ability but also greatly lifts key detection performance, showing high practical utility.

### 3.2 Performance analysis of the VoVGSCSPC-SimAM module

To verify the efficacy of the proposed Slim-Neck integrated with the SimAM module for object detection, ablation experiments were conducted using YOLOv11n as the baseline model. We set up three experiment plans: the original YOLOv11n, YOLOv11n-VoVGSCSPC, and YOLOv11n-VoVGSCSPC-SimAM. All other parts were kept the same to guarantee a fair comparison. The experimental results are shown in **Table 3**.

**Table 3:** Comparison of the performance of three module configurations.

Model	Precision (%)	Recall (%)	mAP50 (%)	mAP50-95 (%)	PLOPs (G)	Parameter (M)	Model Size (MB)
YOLOv11n	87.1	86	89	68.1	6.44	2.59	5.23
YOLOv11n-VoVGSCSPC	89.5	85.8	89.3	68	6.38	2.54	5.16
YOLOv11n-VoVGSCSPC-SimAM	89.6	86.1	89.5	69.1	6.38	2.54	5.16

The experimental results demonstrate that model performance varies across different configurations. The introduction of VoVGSCSPC yields noticeable improvements in Precision and mAP50. Furthermore, the YOLOv11n-VoVGSCSPC-SimAM exhibits superior overall detection capabilities. Its metrics—Precision (89.6%), Recall (86.1%), mAP50 (89.5%), and mAP50-95 (69.1%)—all surpass those of the original YOLOv11n and YOLOv11n-VoVGSCSPC, with particularly pronounced gains observed in mAP50-95. Regarding resource consumption, YOLOv11n-VoVGSCSPC-SimAM’s PLQPs (6.38G), Parameter (2.54M), and Model Size (5.16MB) remain identical to YOLOv11n-VoVGSCSPC, imposing no additional computational burden. This result proves that combining the compact neck structure with the SimAM module can effectively strengthen feature focus and feature expression ability without raising resource consumption. Thus, the core detection performance is improved, and the model has high practical application value.

### 3.3 Ablation experiment

To verify the cooperative performance of modules in the YOLOv11n-DualPC-Lite model and evaluate its overall capability in maize leaf disease detection, this study carried out ablation experiments with a homemade dataset. The experimental results are shown in **Table 4**. Experiment 1 was set as the basic control group, using the original YOLOv11n model.

**Table 4:** Results of ablation experiment.

Experiment	Model	mAP50 (%)	PLOPs (G)	Parameter (M)	Model Size (MB)
1	YOLOv11n	89.0	6.44	2.59	5.23
2	YOLOv11n+C2fDualPConv	89.6	6.23	2.55	5.14
3	YOLOv11n+Slim-Neck_SimAM	89.5	6.38	2.54	5.16
4	YOLOv11n+EfficientHead	89.5	5.19	2.27	4.63
5	YOLOv11n+C2fDualPConv+Slim-Neck_SimAM	90.0	6.17	2.52	5.12
6	YOLOv11n+Slim-Neck_SimAM+EfficientHead	88.7	4.88	2.18	4.49
7	YOLOv11n+C2fDualPConv+EfficientHead	89.9	4.97	2.23	4.54
8	YOLOv11n+C2fDualPConv+Slim-Neck_SimAM+EfficientHead	90.9	4.55	2.13	4.41

From the ablation results of individual modules, each component demonstrates differentiated gains in both accuracy and lightweight performance: incorporating C2fDualPConv improves mAP50 by 0.6 percentage points over the baseline model (Experiment 1), while reducing FLOPs by approximately 3.26%, parameters by about 1.54%, and model size by roughly 1.72%. Incorporating Slim-Neck_SimAM yields a 0.5 percentage point improvement in mAP50, alongside a reduction in FLOPs of approximately 0.93%. Parameters and model size decrease by roughly 1.93% and 1.34%, respectively. This approach achieves modest gains in both accuracy and lightweight performance through optimized feature extraction and architecture. After the introduction of EfficientHead, the mAP50 increased by 0.5 percentage points, while the FLOPs decreased by approximately 19.41%. Additionally, both the number of parameters and the model size were significantly reduced by about 12.36% and 11.47%, respectively. This indicates that the module is designed to optimize the detection head’s lightweight characteristics, thereby substantially diminishing the model’s overhead.

The dual-module combination demonstrates varying degrees of adaptability. The C2fDualPConv+Slim-Neck_SimAM pairing achieves a 1.0 percentage point improvement in mAP50 over the original model, while reducing FLOPs by approximately 4.19%. Parameters and model size decrease by roughly 2.70% and 2.10%, respectively. This accuracy gains stems from synergistic feature extraction and propagation between the two modules. The C2fDualPConv+EfficientHead combination achieved a 0.9 percentage point increase in mAP50, with FLOPs reduced by approximately 22.83%. Parameters and model size decreased by approximately 13.90% and 13.20%, respectively, balancing a slight accuracy gain with a substantial reduction in computational cost. However, the Slim-Neck_SimAM+EfficientHead combination, despite reducing FLOPs by approximately 24.22% and decreasing parameters and model size by approximately 15.83% and 14.15%, respectively, saw a 0.3 percentage point decrease in mAP50. This indicates that when module functionalities are mismatched, gains in lightweight performance may be accompanied by a loss of accuracy.

The whole combination of three modules achieves optimal performance, with mAP50 improving by 1.9% over the original model. FLOPs decrease by approximately 29.35%, while parameters and model size are reduced by approximately 17.76% and 15.68%, respectively, achieving a win-win scenario of accuracy and lightweight efficiency. C2fDualPConv enhances feature extraction, Slim-Neck_SimAM optimizes feature propagation, and EfficientHead streamlines detection heads, substantially reducing computational overhead. These components complement each other functionally and coordinate hierarchically, ultimately meeting the requirements for lightweight and high-precision maize leaf disease detection.

### 3.4 YOLOv11n-DualPC-Lite model performance evaluation

To evaluate the effectiveness of the proposed YOLOv11n-DualPC-Lite model for maize leaf disease detection, its performance was compared against several mainstream lightweight object detection models, including YOLOv5n, YOLOv8n, YOLOv10n, YOLOv11n, YOLOv12n, NanoDet-Plus-m-1.5x, SSDLite-MobileNetV3, and RT-DETR-resnet18. The comparison results are shown in **Table 5**.

**Table 5:** Comparison of YOLOv11n-DualPC-Lite’s performance with other models.

Model	Precision (%)	Recall (%)	mAP50 (%)	mAP50-95 (%)	PLOPs (G)	Parameter (M)	Model size (MB)
YOLOv5n	89.9	81.9	87.6	65.2	7.18	2.51	5.06
YOLOv8n	86.2	83.2	87.3	66	8.20	3.01	5.99
YOLOv10n	84.9	86.5	88.8	66.8	8.40	2.71	5.53
YOLOv11n	87.1	86	89	68.1	6.44	2.59	5.23
YOLOv12n	88	86.7	89.5	69.2	6.48	2.57	5.31
NanoDet-Plus-m-1.5x	73.72	79.38	73.72	50.92	2.97	2.44	4.7
SSDLite-MobileNetV3	77	69	71.39	46.4	1.02	3.44	13.4
RT-DETR-resnet18	88.4	83.7	87.9	65.9	30.2	19.03	38.5
YOLOv11n- DualPC-Lite	90.2	87.3	90.9	71.3	4.55	2.13	4.41

YOLOv11n-DualPC-Lite achieves 90.2% Precision, 87.3% Recall, and 90.9% mAP50, outperforming the original YOLOv11n and other mainstream lightweight models. Simultaneously, the model’s parameter count was reduced to 2.13 million, with FLOPs at just 4.55 G and a model size of 4.41 MB, demonstrating significant lightweighting effects. Compared to the baseline YOLOv11n model, YOLOv11n-DualPC-Lite achieves a 3.1% increase in precision, a 1.9% improvement in mAP50, and a 3.2% rise in mAP50-95. while simultaneously reducing the number of parameters by 17.8%, computational load by 29.3%, and model size by 15.7%. This achieves dual optimization of improved accuracy and lightweight design.

Compared to the newer YOLOv12n model, it achieves a 1.4 percentage-point improvement in mAP50 and a 2.1 percentage-point increase in mAP50-95. While maintaining leading detection accuracy, it reduces the number of parameters by 17.1% and the computational load by 29.8%, demonstrating more substantial lightweight advantages. Compared to NanoDet-Plus-m-1.5x and SSDLite-MobileNetV3, YOLOv11n-DualPC-Lite achieves mAP50 scores 17.18% and 19.51% higher, respectively, and mAP50–95 scores 20.38% and 24.9% higher, respectively, demonstrating significant performance advantages. Additionally, its model size is only 4.41MB, significantly smaller than RT-DETR-ResNet18’s 38.5MB, making it more suitable for lightweight deployment scenarios such as edge devices.

The experimental results above show that YOLOv11n-DualPC-Lite cuts down model complexity effectively while keeping high detection accuracy. It achieves a good balance between detection performance and lightweight deployment needs, thus being more suited to the practical requirements of real-time field detection of maize leaf diseases.

### 3.5 Visualization analysis

**Figure 12:**
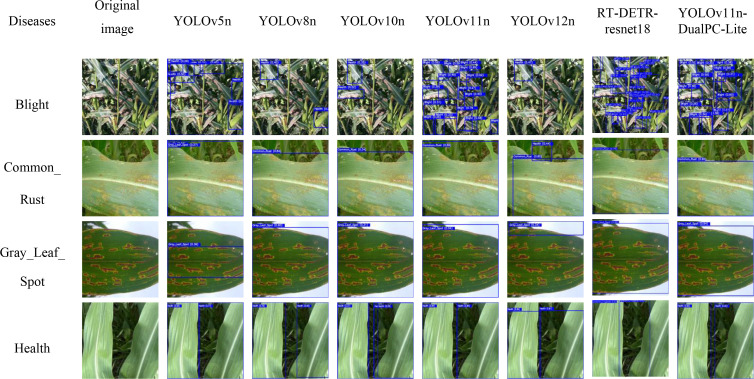
Prediction maps for different models.

**Figure 13:**
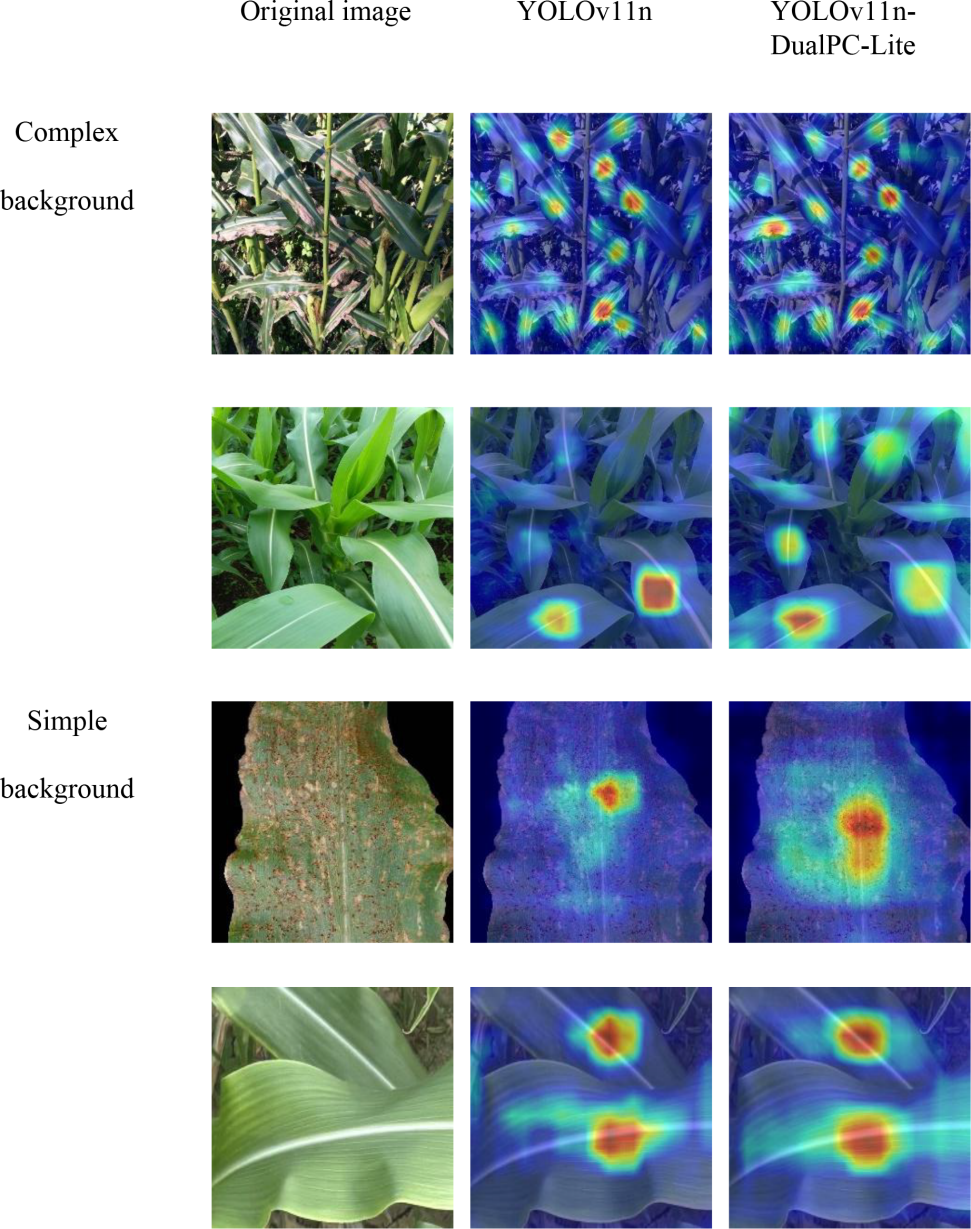
Visual heatmap.

This study selected one representative sample each of maize leaf Blight, Common_Rust, Gray_Leaf_Spot, and Health. Disease detection predictions were conducted using the YOLOv5n, YOLOv8n, YOLOv10n, YOLOv11n, YOLOv12n,RT-DETR-resnet18, and YOLOv11n-DualPC-Lite models. The results are presented in **Figure 12**. To further examine the detection advantages of the YOLOv11n-DualPC-Lite model proposed in this study, we employ the gradient weighted category activation map (GradCAM) for visual analysis. GradCAM computes the gradient of the final convolutional layer’s feature map with respect to the target category via backpropagation. This weighted information is then used to generate a heatmap that intuitively highlights the key visual areas the model focuses on during prediction. The results are shown in **Figure 13**.

As shown in the model prediction images, for the lightweight object detection tasks on maize leaf blight, common rust, gray leaf spot, and health, YOLOv11n-DualPC-Lite demonstrates significantly superior detection accuracy and confidence compared to six mainstream models: YOLOv5n, YOLOv8n, YOLOv10n, YOLOv11n, YOLOv12n, and RT-DETR-resnet18. It achieved high confidence levels of 0.90 and 0.94 for common rust and gray leaf spot detection, respectively. For healthy leaves, it delivered two stable high-confidence predictions of 0.92, both at the highest confidence level. In complex blight disease scenarios, although fewer disease regions were identified compared to YOLOv11n and RT-DETR-ResNet18, the average confidence level was higher, and the number of misclassifications was lower. Visualization of GradCAM heatmaps reveals that in complex backgrounds, YOLOv11n-DualPC-Lite effectively focuses attention on the leaf’s main body. The warmer color tones of the main body exhibit higher brightness, creating a more pronounced contrast with the background colors and resulting in a cleaner background. In simple backgrounds, such as typical rust-affected leaf scenarios, YOLOv11n’s heatmap exhibits warm tones only in the localized lesion areas with limited coverage. In contrast, the heatmap of YOLOv11n-DualPC-Lite shows brighter warm colors and covers larger areas of dense lesions, capturing the key visual traits of diseases more thoroughly. On samples of healthy leaves with clear texture, YOLOv11n’s heatmap focuses only on localized areas. Conversely, YOLOv11n-DualPC-Lite’s heatmap not only exhibits greater brightness but also covers most of the leaf’s main body, enabling more comprehensive extraction of target features. This visualization elucidates the core reason for YOLOv11n-DualPC-Lite’s superior detection performance at the feature extraction level: it efficiently captures key visual characteristics of the target while filtering out irrelevant background information.

### 3.6 Maize leaf disease detection system construction

The Raspberry Pi main control unit serves as the core component of the system, running the YOLO algorithm to detect and identify maize leaf diseases from video streams captured by the camera. The camera connects via the Raspberry Pi’s USB port, enabling real-time video data capture. The system is powered by an independent power supply to ensure the portability of the entire setup. The trained YOLOv11n-DualPC-Lite model is deployed on the Raspberry Pi for execution.

#### 3.6.1 System introduction

**Figure 14:**
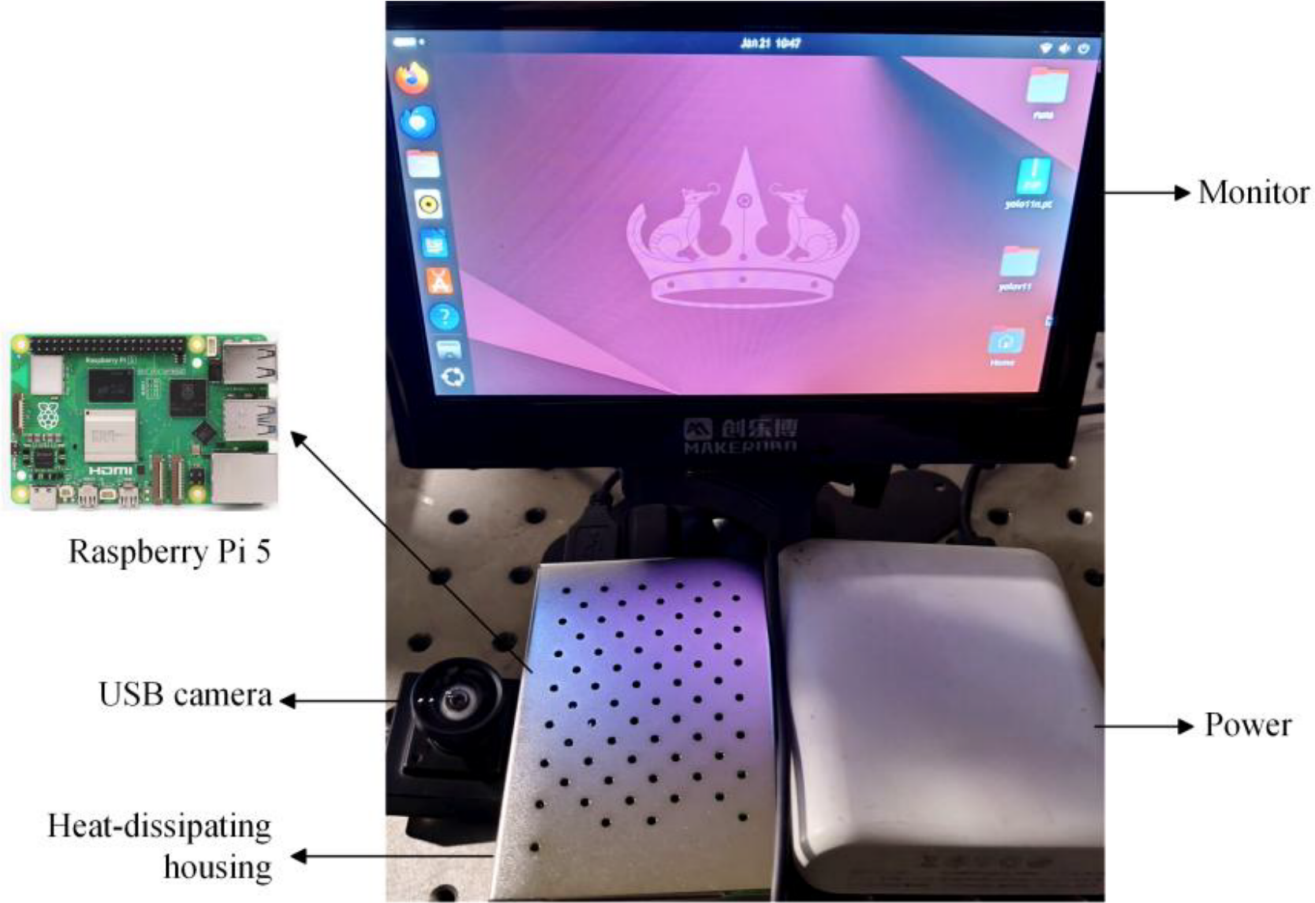
System hardware components.

The system’s main controller utilizes a Raspberry Pi 5 (8GB edition) equipped with the BCM2712 chip, offering native edge computing capabilities to independently handle control scheduling and local data processing. Measuring just 85mm × 56mm, this development board utilizes a quad-core ARM Cortex-A76 processor and VideoCore VII GPU, delivering performance comparable to entry-level x86 devices. It balances low power consumption with strong scalability, making it suitable for IoT, edge computing, and machine vision applications. Within this system, a JR GW200 USB camera with 1920×1080 resolution captures video and transmits it to the Raspberry Pi. The YOLOv11n model then performs real-time recognition and inference directly. The hardware configuration is illustrated in **Figure 14**.

#### 3.6.2 Deployment and operation of the detection system

**Figure 15:**
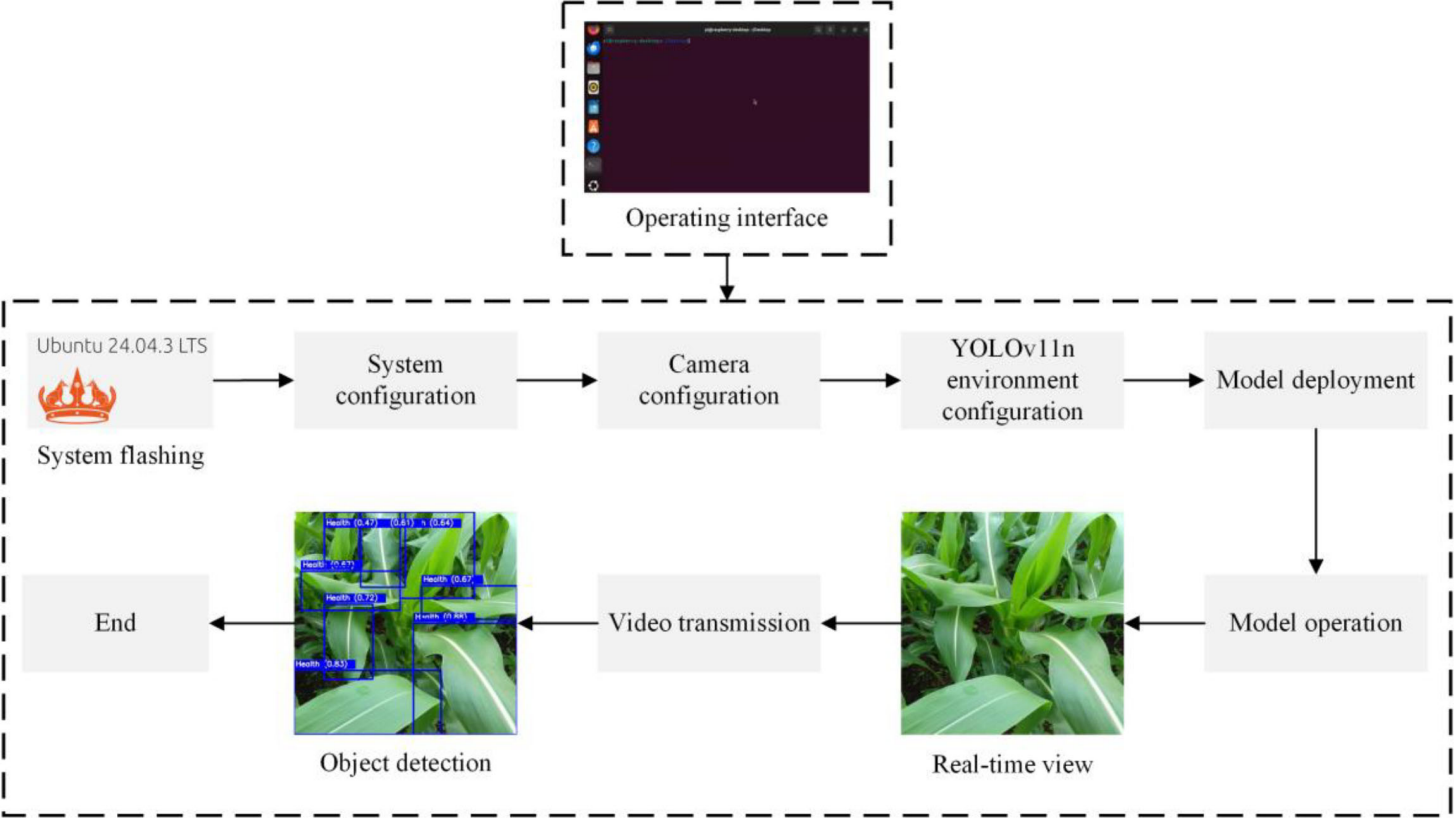
System deployment and operational workflow.

The latest Ubuntu 22.04.03 operating system, obtained from the official Raspberry Pi website, was selected and installed on the Raspberry Pi 5. Following system installation, the terminal command was opened to configure system settings. The USB camera source was identified and confirmed as 0. Downloaded the YOLOv11n code onto the Raspberry Pi system and configured the necessary environment. Uploaded the trained model files to the Raspberry Pi, adjusted parameter settings, and related configurations. Entered the terminal command to run the program; once the camera was activated, the system commenced capturing and recognizing images. **Figure 15** illustrates the system deployment and operational workflow.

**Figure 16:**
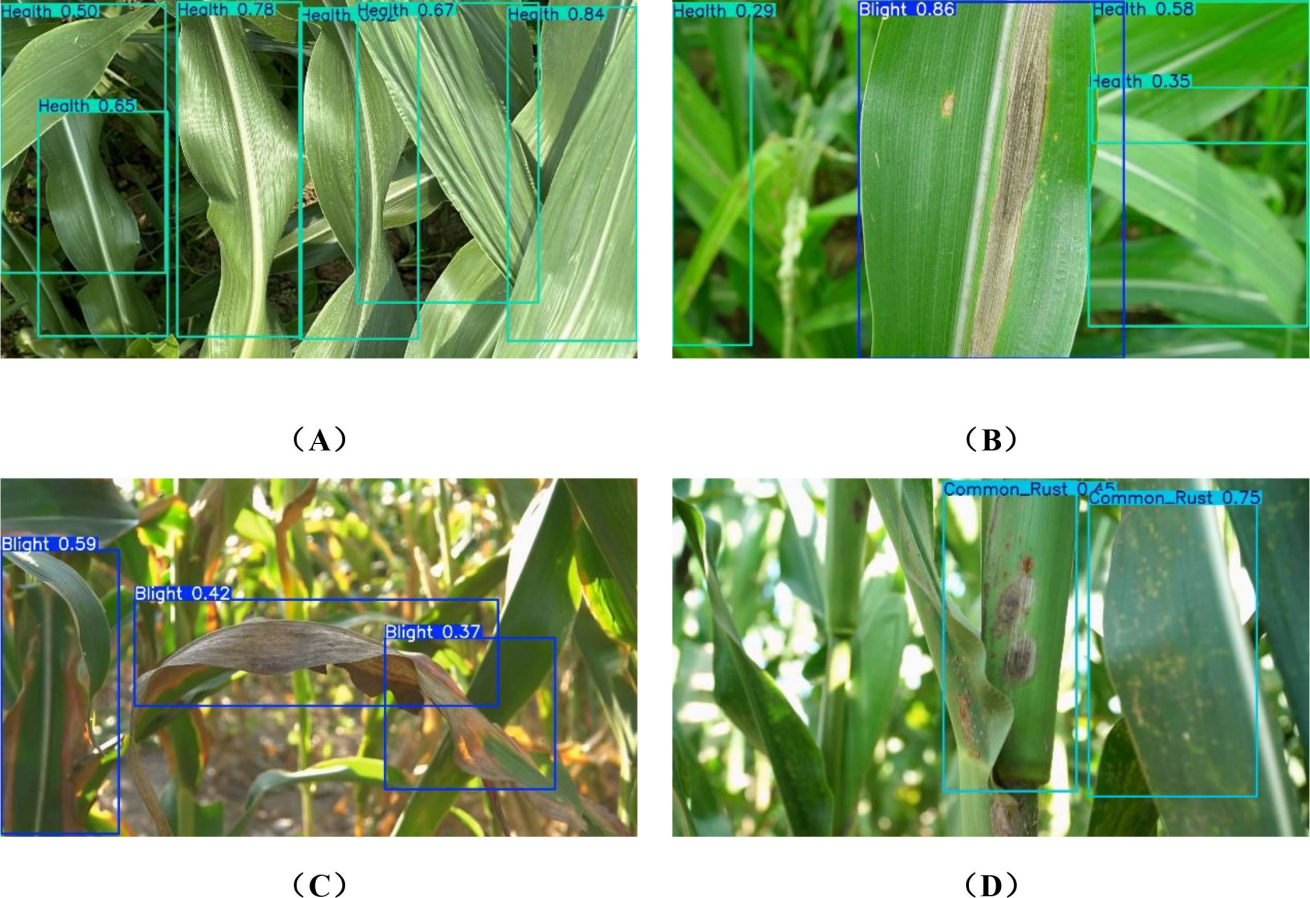
Videos detected by YOLOv11n-DualPC-Lite.

To evaluate the performance of deployed models, both the original YOLOv11n and the improved YOLOv11n-DualPC-Lite were tested on Raspberry Pi 5 hardware. Two video clips recorded with a mobile phone in a maize field were selected for testing. These MP4 files had a total duration of 180 seconds and an original resolution of 1920×1080 pixels. Copy the video to the Raspberry Pi. Before inputting the video into the model, YOLOv11 performs preprocessing: first resizing the image to 640×640 pixels via letterboxing, then normalizing the pixel values. Detection results are compared as shown in **Table 6** and **Figure 16**.

**Table 6:** Model deployment performance comparison.

Model	PLOPs (G)	Parameter (M)	Model size (MB)	Frame rate (FPS)
YOLOv11n	6.44	2.59	5.23	1.8
YOLOv11n-DualPC-Lite	4.55	2.13	4.41	2.3

The YOLOv11n-DualPC-Lite model features low computing load and small memory usage, making it ideal for low-resource edge devices. It also shows clear speed benefits on the Raspberry Pi 5, raising the frame rate from 1.8 frames per second (FPS) to 2.3 FPS—a 27.8% increase. The model balances fast running speed and improved detection precision, recognizing more disease spots. This better meets the dual needs of agricultural applications for real-time response and accuracy. The progression of maize leaf diseases is relatively slow, allowing periodic inspections to effectively monitor disease occurrence and spread. Therefore, a frame rate of 2.3 FPS demonstrates high practical value in this scenario, providing reliable technical support for disease detection in agricultural settings.

## 4 Discussion and conclusion

### 4.1 Discussion

In existing maize leaf disease detection research, traditional machine learning relies on manually designed features, resulting in poor generalization capabilities. Although two-stage object detection models such as Faster R-CNN deliver high detection accuracy, they require massive computational resources, making it difficult to achieve real-time detection on resource-constrained edge devices. n the model comparison experiment, the lightweight SSD and RT-DETR models were much larger than the YOLO lightweight series in terms of model size, and their detection accuracy was lower than that of the YOLOv11n-DualPC-Lite model.

The proposed YOLOv11n-DualPC-Lite model achieves improved detection accuracy while reducing computational complexity and parameter count through the synergistic optimization of the C2fDualPConv module, Slim-Neck_SimAM architecture, and EfficientHead detection head. Successfully deployed on Raspberry Pi 5. The portable Raspberry Pi-based recognition system is adapted for field inspection scenarios, providing a viable technical solution for real-time prevention and control of maize leaf diseases. Its design approach also offers technical reference for lightweight improvements in pest and disease detection models for other crops.

However, the long-term operational stability of the Raspberry Pi system in complex field environments still requires further validation. Although the detection frame rate on the Raspberry Pi 5 has significantly improved compared to the original model, reaching 2.3 FPS, it may still be insufficient for highly dynamic scenes. Future efforts will explore diverse model deployment methods and high-performance edge computing devices to further enhance the overall performance of the detection system.

### 4.2 Conclusion

This paper addresses key challenges in real-time maize leaf disease detection, including balancing model lightweighting with accuracy and addressing resource constraints on edge devices. It proposes an improved YOLOv11n-DualPC-Lite model. The C2fDualPConv module uses partial convolution to cut down unnecessary calculations and improve detailed feature extraction. The Slim-Neck_SimAM structure uses the SimAM attention method to focus more on key features without adding extra parameters. The EfficientHead balances detection accuracy and computing cost.

By analyzing maize leaf disease prediction maps and GradCAM thermal maps, this model shows obvious advantages in detection accuracy, focus on key features and filtering background noise. It also explains why its feature extraction ability is improved. Precision, recall rate and mAP50 reached 90.2%, 87.3% and 90.9% respectively, proving the model’s excellence. Compared with the original YOLOv11n, this model has 17.8% fewer parameters and 29.3% less computing cost. On Raspberry Pi 5, its detection speed is increased to 2.3 FPS, 27.8% higher than the basic model. Its performance is much better than mainstream lightweight models like YOLOv5n and YOLOv12n, meeting the actual demand for real-time detection on on-site devices in maize fields.

Future research efforts will focus on the following areas: First, constructing a maize leaf disease sample dataset covering diverse climates, varieties, and scenarios. Second, enhancing the device coordination capabilities and environmental adaptability of the detection system. Third, further optimizing model lightweighting and acceleration techniques to improve real-time performance and practical value.

## Data Availability

The original contributions presented in the study are included in the article/supplementary material. Further inquiries can be directed to the corresponding author.
